# Targeting the JNK Gatekeepers: Structural Evolution and Medicinal Chemistry of MKK4 and MKK7 Inhibitors

**DOI:** 10.3390/molecules31040672

**Published:** 2026-02-15

**Authors:** Min Zhao, Baojian Li, Ying Gao, Yan Liang, Nanqi Shao, Xinbo Shi, Jie Li

**Affiliations:** 1 School of Pharmacy, Xinjiang Second Medical College, Karamay 834000, China; 18232189381@163.com (M.Z.); lbj9591@163.com (B.L.); 18829190996@163.com (Y.G.); liangyan@xjsmc.edu.cn (Y.L.); shaonanqi2024@126.com (N.S.); 2 Science and Innovation Center, Xinjiang Second Medical College, Karamay 834000, China; 3 Shaanxi Collaborative Innovation Center of Chinese Medicine Resources Industrialization, Shaanxi University of Chinese Medicine, Xianyang 712046, China

**Keywords:** MKK4 (MAP2K4), MKK7 (MAP2K7), JNK signaling, kinase inhibitors, liver regeneration, covalent inhibitors, medicinal chemistry, fibrosis, HRX215

## Abstract

The c-Jun N-terminal kinase (JNK) pathway is a central driver of fibrosis, inflammation, and neurodegeneration. While direct JNK inhibitors have shown therapeutic promise, achieving high isoform selectivity remains a significant medicinal chemistry challenge. Furthermore, targeting the upstream ‘gatekeepers’ MKK4 and MKK7 offers a distinct mechanism to modulate pathway output with greater precision. Consequently, medicinal chemistry efforts have shifted upstream to the dual-specificity kinases MKK4 and MKK7. This review critically evaluates the structural biology and pharmacological evolution of small-molecule inhibitors targeting these nodes. We contrast the distinct therapeutic landscapes of the two kinases: while MKK4 inhibition has emerged as a breakthrough strategy for unlocking liver regeneration (exemplified by the first-in-class clinical candidate HRX215), MKK7 inhibition is primarily pursued for its anti-fibrotic and anti-inflammatory potential. Special attention is given to structure-based design strategies, including the exploitation of the unique hinge-region cysteine (Cys218) for MKK7-specific covalent targeting and the optimization of scaffold selectivity against off-targets like BRAF. Finally, we discuss emerging modalities, such as PROTACs and dual inhibitors, outlining a roadmap for the next generation of precision therapeutics targeting the MKK–JNK axis.

## 1. Introduction

The mitogen-activated protein kinase (MAPK) signaling cascades serve as central integration points for transducing extracellular stimuli into specific cellular responses, governing fundamental processes such as proliferation, differentiation, apoptosis, and the inflammatory response [1,2]. Among these pathways, the c-Jun N-terminal kinase (JNK) signaling axis—often referred to as the stress-activated protein kinase pathway—plays a pivotal role in the pathogenesis of diverse human disorders, including neurodegenerative diseases, fibrotic conditions, inflammatory disorders, and cancer [3,4,5,6,7]. While the dysregulation of JNK signaling is a well-established driver of disease, the therapeutic validation of this pathway has historically proven to be challenging. Although recent advances have led to the identification of isoform-selective JNK inhibitors with reduced toxicity, targeting the highly conserved ATP-binding cleft of JNKs remains pharmacologically challenging. Moreover, direct blockade of JNKs may not always offer the subtle signal modulation required for complex diseases [8,9,10].

To overcome the limitations associated with direct JNK targeting, medicinal chemistry efforts have increasingly shifted upstream to the “gatekeepers” of the pathway: the mitogen-activated protein kinase kinases (MAP2Ks), specifically MKK4 (MAP2K4/SEK1) and MKK7 (MAP2K7) [11,12]. These dual-specificity kinases represent the convergence point of the three-tiered kinase cascade, linking diverse upstream MAP kinase kinase kinases (MAP3Ks) to the downstream JNK effectors. MKK4 and MKK7 are responsible for the dual phosphorylation of the Thr-Pro-Tyr motif within the activation loop of JNKs, a requisite step for full enzymatic activation [13,14,15].

Crucially, MKK4 and MKK7 are not functionally redundant, offering distinct therapeutic opportunities. MKK7 functions as a specific activator of JNKs, mediating inflammatory and stress responses, making it an attractive target for inflammatory and autoimmune indications [16,17,18]. In contrast, MKK4 exhibits a broader substrate profile, capable of activating both the JNK and p38 MAPK pathways [19,20]. Recent pharmacological and genetic studies have highlighted a unique role for MKK4 inhibition in promoting hepatocyte proliferation and liver regeneration, sparking renewed interest in this kinase as a regenerative medicine target [21,22]. This divergence in biological function underscores the necessity for highly selective chemical probes that can discriminate not only between MKK4 and MKK7 but also against other closely related MAP2Ks.

Despite their therapeutic promise, the development of potent and selective small-molecule inhibitors for MKK4 and MKK7 has lagged behind that of downstream MAPKs. However, recent advances in structural biology and fragment-based drug design have led to the identification of novel chemotypes and binding modes, ranging from classical ATP-competitive inhibitors to covalent modifiers targeting non-catalytic cysteines [11].

This review aims to provide a comprehensive analysis of the current landscape of MKK4 and MKK7 inhibitors. We will discuss the structural characteristics of these kinases that facilitate ligand binding, examine the medicinal chemistry strategies employed to improve potency and selectivity—including the evolution of key chemical scaffolds—and critically evaluate the therapeutic potential of targeting these upstream kinases in the context of liver disease, oncology, and inflammation. By synthesizing data from structural studies and pharmacological evaluations, we highlight the remaining challenges and future directions for drugging these critical nodes of the JNK signaling architecture.

## 2. The Biological Functions and Therapeutic Potential of MKK4/7

### 2.1. The JNK Signaling Architecture

**Figure 1:**
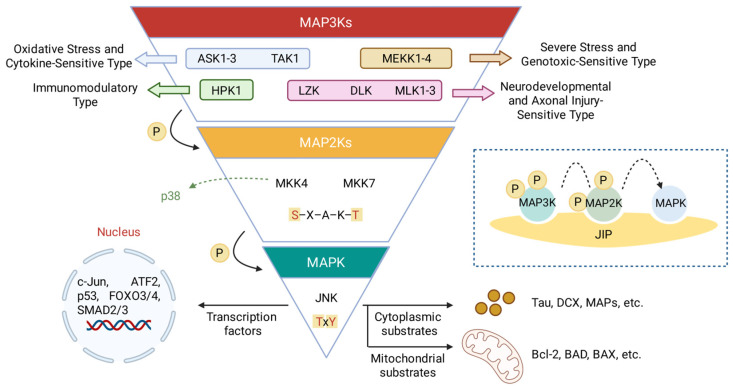
Schematic representation of the JNK signaling architecture. The pathway exhibits a funnel-like topology where diverse upstream stimuli converge upon the central gatekeepers, MKK4 and MKK7. The conserved phosphorylation motif (S–X–A–K–T in MKKs) is highlighted. The dashed inset illustrates the mechanism of spatiotemporal regulation mediated by scaffold proteins (e.g., JIPs). By tethering specific MAP3K, MAP2K, and MAPK components in close proximity, these scaffolds facilitate efficient sequential phosphorylation and ensure signal specificity. The dashed arrow from MKK4 indicates its potential crosstalk with the p38 pathway.

The JNK pathway represents an evolutionarily conserved signaling cascade that orchestrates cellular responses to environmental stress, pro-inflammatory cytokines, and growth factors. Functioning within the canonical three-tiered MAPK module, the signal transmission is initiated by a diverse group of MAP3Ks (Table 1), such as ASK1, TAK1, and MEKK1, which sense upstream stimuli and phosphorylate the activation loops of the dual-specificity kinases, MKK4 and MKK7 (Figure 1) [23,24,25]. MKK4 and MKK7 share a kinase motif known as Ser–Xaa–Ala–Lys–Thr (S–X–A–K–T), which is located in the T-loop of the kinase domain [26]. Phosphorylation of both hydroxy residues (MKK4: Ser257 and Thr261; MKK7: Ser271 and Thr275) is required for the full activation of both proteins [27,28]. Studies have also demonstrated that Ser257 phosphorylation is essential, and Thr261 phosphorylation is required for full MKK4 activity [29].

As the central nodes of this cascade, MKK4 and MKK7 serve as the exclusive activators of JNK proteins (JNK1, JNK2, and JNK3). Activation occurs via dual phosphorylation of the conserved Threonine–Proline–Tyrosine (TxY) motif located within the JNK activation loop [13,14,15]. Importantly, while both MKK4 and MKK7 can mediate dual phosphorylation of JNKs in vitro, this process is inefficient individually, as MKK4 exhibits a striking preference for the tyrosine residue and MKK7 for the threonine residue, suggesting that both kinases are required for efficient dual phosphorylation in cells [30,31]. The dual phosphorylation event is estimated to induce a conformational change in the JNK activation loop, which creates a functional active site by realigning the N- and C-terminal domains, thus stabilizing the kinase in a conformation suitable for substrate binding [32,33]. Once activated, JNKs translocate to the nucleus or target mitochondrial proteins to phosphorylate specific substrates, most notably c-Jun, a component of the AP-1 transcription factor complex. This phosphorylation event (e.g., at Ser63 and Ser73 of c-Jun) drives the transcriptional expression of genes regulating apoptosis, survival, and cellular differentiation [33,34].

Crucially, the architecture of this pathway exhibits an “hourglass” topology: while there are over a dozen upstream MAP3Ks and multiple downstream JNK isoforms and substrates, the signal must funnel through the bottleneck of just two MAP2Ks (MKK4 and MKK7). This structural feature highlights MKK4 and MKK7 as strategic intervention points for therapeutically modulating JNK signaling output without engaging the complex redundancy found at the MAP3K level.

While MKK4 and MKK7 are capable of activating all JNK isoforms (JNK1, JNK2, and JNK3) in vitro, the precise differential regulation observed in vivo is orchestrated primarily by scaffold proteins and subcellular compartmentalization. Scaffold proteins, such as the JNK-interacting proteins (JIP1, JIP2, JIP3/JSAP1) and plenty of SH3 (POSH), act as molecular platforms that assemble specific MAP3K–MAP2K–MAPK modules. For example, JIP3 has been shown to preferentially bind MKK7 and JNK3, facilitating signal transduction specifically in neuronal contexts [35]. Conversely, JIP1 can coordinate MKK7–JNK1 signaling in response to metabolic stress [36]. This scaffolding mechanism ensures that although MKK4 and MKK7 share overlapping substrates, their signaling output is spatially restricted and kinetically efficient, allowing for distinct biological outcomes (e.g., JNK1-mediated proliferation vs. JNK3-mediated apoptosis) depending on the available scaffold repertoire in a given tissue.

### 2.2. Biological Functions and Therapeutic Potential

The validation of MKK4 and MKK7 as therapeutic targets is grounded in their non-redundant physiological roles and their involvement in distinct pathological signaling axes. While both kinases serve as the proximal activators of JNK, their contributions to embryonic development, tissue homeostasis, and disease progression are markedly different.

#### 2.2.1. MKK4 in Liver Regeneration and Hepatoprotection

**Figure 2:**
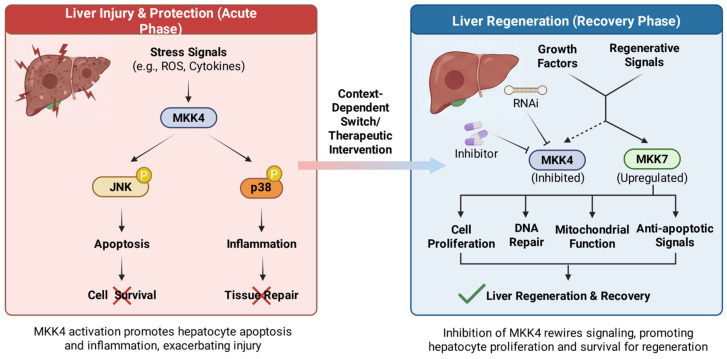
The role of MKK4 in liver protection and regeneration.

Liver regeneration is a complex, orchestrated process where hepatocytes re-enter the cell cycle to restore functional liver mass following injury or resection. While the liver possesses a unique innate regenerative capacity, this process is often blunted in severe acute liver failure (ALF) or chronic liver diseases (CLDs) [37,38]. Recent functional genetic screens utilizing in vivo RNA interference (RNAi) have identified MKK4 as a master negative regulator [39]. Under both physiological and stress conditions, MKK4 limits the extent of hepatocyte proliferation, while silencing or pharmacological inhibition of MKK4 releases this brake, resulting in a robust increase in hepatocyte DNA synthesis, faster cell cycle entry, and enhanced overall regenerative capacity (Figure 2). The mechanism by which MKK4 inhibition promotes regeneration is counterintuitive, as JNK signaling is traditionally associated with apoptosis. The current prevailing model involves a “rerouting” of stress signaling. When MKK4 activity is inhibited, upstream stress signals (from cytokines like TNF-α or growth factors) can no longer flow through MKK4. Consequently, the signaling flux is rerouted predominantly through MKK7. This MKK7-driven axis preferentially activates JNK1. Unlike the apoptotic AP-1 complex often driven by MKK4, the specific MKK7–JNK1 axis leads to the phosphorylation and activation of distinct transcription factors, most notably activating transcription factor 2 (ATF2) and ELK1. These transcription factors drive the expression of pro-proliferative genes (e.g., *Ccnd1* encoding Cyclin D1) and survival factors, thereby accelerating the G1/S phase transition in hepatocytes [20,40]. Moreover, suppression of MKK4 expression under steady-state conditions confers resistance to Fas-mediated apoptosis in hepatocytes [41].

#### 2.2.2. The Role of MKK7 in Cancer

**Figure 3:**
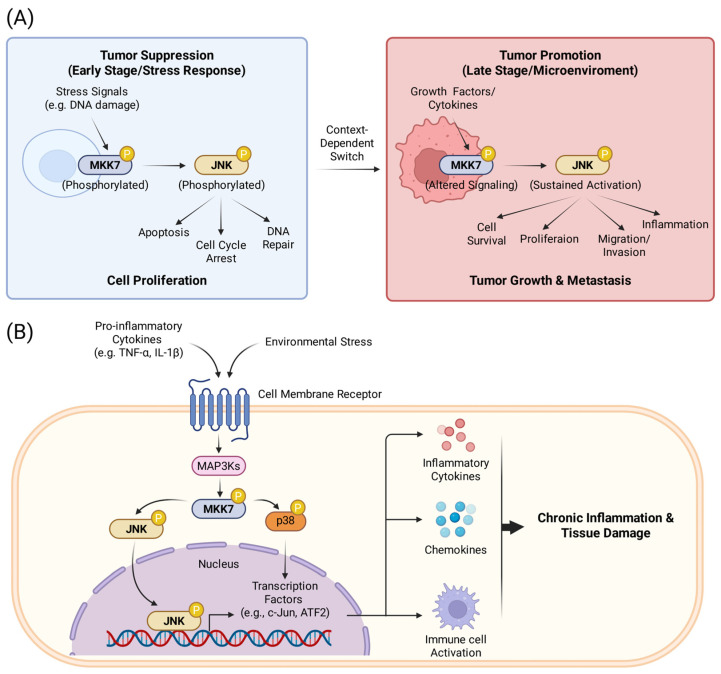
(**A**) The dual role of MKK7 in cancer progression. (**B**) Schematic representation of the MKK7 signaling pathway in the inflammatory response.

The role of MKK7 in tumorigenesis is highly context-dependent, functioning as a tumor suppressor in early-stage development and genomic stability maintenance, while acting as a pro-tumorigenic or pro-metastatic factor in established malignancies and specific genetic contexts (Figure 3A). As a tumor suppressor, MKK7 is essential for maintaining genomic stability and facilitating stress-induced senescence. In *KRasG12D*-driven lung carcinomas and *NeuT*-driven mammary tumors, MKK7 senses oncogenic stress and promotes p53 phosphorylation and stability via the JNK signaling axis. Loss of MKK7 leads to p53 instability, thereby accelerating tumor onset and reducing overall survival [42]. Furthermore, MKK7 functions cooperatively with p53 to regulate Polo-like kinase 4 (PLK4) activity, which prevents centrosome overduplication during mitosis and protects cells from aneuploidy, a hallmark of cancer genomic instability [43]. In prostate cancer models, MKK7 deficiency has also been shown to synergize with PTEN loss, driving the rapid development of invasive adenocarcinoma [44,45].

However, the role of MKK7 is double-edged. In established malignancies and specific genetic contexts, MKK7 acts as a pro-tumorigenic or pro-metastatic factor. In T-cell acute lymphoblastic leukemia (T-ALL), the transcription factor Kruppel-like factor 4 (KLF4) typically represses the *MAP2K7* gene. However, the loss of KLF4 (often via DNA methylation) results in the overexpression of MKK7, which drives excessive JNK pathway activation and leukemic cell proliferation [46]. In the context of metastasis, a specific rare polymorphism in MKK7 (p.Glu116Lys) found in lung cancer patients is significantly associated with increased metastatic potential. Mechanistically, this mutation alters the expression of cancer-associated genes, specifically upregulating *STC2*, *SLC1A3*, and *BCL10*, while downregulating *SAA1* and *CDH5* [47]. Additionally, in colorectal cancer, MKK7 is a direct target of the metastasis-suppressor miR-493, which binds to the 3′-UTR of MKK7 mRNA. Loss of this regulatory interaction leads to MKK7 upregulation, thereby promoting liver metastasis [48].

The activity of MKK7 is further modulated by specific protein–protein interactions and post-translational modifications that cancer cells exploit to evade apoptosis. In multiple myeloma, the NF-β pathway upregulates GADD45β, which physically binds to MKK7 and blocks ATP access to its catalytic pocket, thereby inhibiting the MKK7–JNK apoptotic axis and promoting cell survival [49,50]. Similarly, in HCC, the TOR signaling regulator TIPRL binds to MKK7 and recruits the phosphatase PP2Ac to form a complex that facilitates MKK7 dephosphorylation. This inactivation of MKK7 contributes to resistance against TRAIL-induced apoptosis [51]. MKK7 activity in breast cancer is also suppressed by neddylation mediated by the E3 ligase RanBP2, where RanBP2 knockdown has been shown to restore MKK7 kinase activity and inhibit cell proliferation [52]. Other regulatory interactions include RACK1, which acts as a scaffold to enhance MKK7 activity in HCC, and RASSF7, which binds specifically to phosphorylated MKK7 to potentially hinder its interaction with downstream substrates, thereby negatively regulating pro-apoptotic signaling [53,54].

#### 2.2.3. The Role of MKK7 in Inflammation

MKK7 plays a pivotal role in the innate immune system, acting as a central regulator of macrophage biology and the inflammatory response (Figure 3B). Upon stimulation by pathogens or pro-inflammatory cytokines, MKK7 is indispensable for the production of key inflammatory cytokines, including TNF-α, IL-1α, IL-1β, and IL-6, particularly in response to bacterial lipopolysaccharides (LPS). While the related kinase MKK4 also contributes to this signaling network, its influence on cytokine production is modest compared to the dominant role of MKK7. Beyond cytokine secretion, MKK7 governs the physical mobilization of immune cells. Deficiency of MKK7 leads to a significant blockade in macrophage migration and invasion capabilities. Furthermore, MKK7 is essential for macrophage polarization towards the M1 pro-inflammatory phenotype, thereby orchestrating the initiation of an effective immune defense [17,55].

Mechanistically, MKK7 functions through both canonical and non-canonical signaling pathways to execute these inflammatory responses. Classically, MKK7 is known as the specific upstream activator of the JNK pathway, which drives the transcriptional activation of cytokine genes. However, recent evidence indicates a critical crosstalk mechanism where MKK7 is also required for the full activation of p38 MAPK in macrophages, challenging the conventional understanding that MKK3/6 are the sole activators of p38 [55]. This dual regulation of both JNK and p38 pathways suggests that MKK7 acts as a master node in the inflammatory signaling network. Additionally, the activation of this pathway often relies on the coordination of scaffold proteins, such as JIP1, which facilitate the interaction between MKK7 and its upstream or downstream partners to ensure robust signaling [36].

The regulatory power of MKK7 makes it a significant contributor to the pathology of chronic inflammatory diseases such as rheumatoid arthritis (RA) and sepsis. In the context of RA, MKK7 drives joint destruction not only by promoting cytokine release but also by regulating matrix metalloproteinases (MMPs), the enzymes responsible for degrading the extracellular matrix. Consequently, therapeutic strategies targeting MKK7 have shown promise. For instance, the inhibition of MKK7 using antisense oligonucleotides (ASOs) has been demonstrated to decrease disease severity in mouse models of serum transfer arthritis [56]. These findings highlight MKK7 as a promising therapeutic target for managing uncontrolled inflammatory responses, preventing the transition from acute to chronic inflammation, and mitigating tissue damage in autoimmune conditions.

## 3. Structures of MKK4 and MKK7

**Figure 4:**
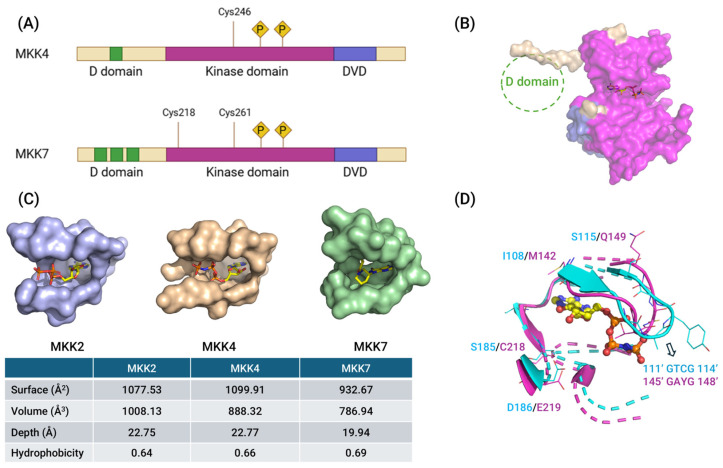
(**A**) Schematic illustration of MKK4 and MKK7. The two kinases contain several functional domains for activation and binding of other proteins (D, docking domain; DVD, domain of versatile docking; P, phosphorylation site). They are activated by phosphorylation of two residues in their kinase domain (MKK4: Ser257 and Thr261; MKK7: Ser271 and Thr275). Cysteine residues for covalent binding are also highlighted. (**B**) Crystal structure of human non-phosphorylated MKK4 kinase domain complexed with AMP-PNP (PDB: 3ALN). The kinase domain is colored in magenta, and the DVD domain is colored in light blue. However, the currently known crystal structures of MKK4 and MKK7 are missing the D domain in the N-lobe. (**C**) Three-dimensional structural models of the ATP-binding pockets of MKK2 (purple), MKK4 (wheat), and MKK7 (green) are displayed. Quantitative characterization of the ATP-binding pockets, including surface area (Å^2^), volume (Å^3^), depth (Å), and hydrophobicity, is computed using ProteinPlus [71]. The corresponding data are summarized in the table below. (**D**) The ATP-binding pockets of MKK4 (cyan, PDB: 3ALN) and MKK7 (magenta, PDB: 6QFT) are superimposed, with key divergent residues highlighted. Corresponding amino acid pairs are labeled: S115/Q149, I108/M142, S185/C218, and D186/E219. Sequence motifs in the entrance of ATP-binding pocket are also indicated: GTCG (positions 111′–114′) in MKK4 and GAYG (positions 145′–148′) in MKK7. The motif of MKK7 points inward toward the binding pocket, while that of MKK4 extends outward, which may explain the small volume and shallow depth of the ATP-binding pocket in MKK7.

MKK4 and MKK7 proteins are encoded in humans by the *MAP2K4* (on chromosome 17p12 with 11 exons) and *MAP2K7* (on chromosome 19p13 with 12 exons) genes. In humans, alternative splicing of *MAP2K4* gives rise to two isoforms: isoform 2, the longer variant, includes an additional in-frame exon in the 5′ coding region compared to isoform 1. In mice, alternative splicing of the *MAP2K7* gene produces a set of protein isoforms with distinct N-terminal (α, β, γ) and C-terminal (1, 2) variants. These murine isoforms have predicted molecular weights ranging from 38 to 52 kDa and consist of approximately 345 to 467 amino acid residues [17,57,58]. Both MKK4 and MKK7 exhibit the canonical structural architecture of the MAP2K subfamily and share 54% sequence identity. Their three-dimensional structures fold into two distinct lobes: a smaller N-lobe, comprising five β-sheets and one α-helix, and a larger C-lobe primarily composed of α-helices. A flexible segment connecting the two lobes houses the ATP-binding site. Functionally, MKK proteins are organized into three primary parts (Figure 4A,B): the N-terminal docking domain (D domain), the central kinase domain, and the C-terminal domain for versatile docking (DVD) [17,20,59].

The D domain mediates interactions with downstream substrates, particularly JNK, and also serves as a binding site for scaffold proteins such as Arrestin-1 and -2 (in the case of MKK4) [60]. Structurally, the D domains of MKK4 and MKK7 consist of a cluster of two to three basic residues, followed by a short spacer (1–2 residues) and a hydrophobic–X–hydrophobic motif [58,61]. Both MKK4 and MKK7 interact with JNK through their D domains, but they differ in valency. The three MKK7 splice variants exhibit differential activity toward JNK: MKK7α displays lower activity compared to β and γ isoforms. This difference likely arises because the β and γ isoforms contain three D domains that bind to the JNK DRS (Figure 4A), whereas the α isoform possesses only a single D domain [57]. Similarly, MKK4 engages JNK through a single N-terminal D domain.

The central ATP-binding pocket is highly conserved across the MAP2K family, yet MKK4 and MKK7 possess distinct characteristics that confer functional specificity (Figure 4D). For example, the MKK4 hinge region specifically contains neutral residues Leu180 and Ser182, unlike homologous positions in MKK2, which contain charged residues [62]. MKK7 has the smallest volume and a significantly shallower depth among all MEK isoforms with available crystal structures (Figure 4C). Its active site is characterized by a high proportion of hydrophobic residues and fewer charged residues compared to MKK1/2/4/6. While MKK1, MKK2, MKK4, and MKK6 binding cavities are predominantly negatively charged, the MKK7 cavity exhibits patches of partial positive and negative charges [63]. Both MKK4 and MKK7 possess a highly conserved cysteine located immediately before the DFG motif (Cys246 in MKK4 and Cys261 in MKK7). However, unlike other MEK family members, MKK7 is unique in containing a Cys218 in the hinge region, a position where only 11 human kinases share an equivalent cysteine [64].

MKK4 and MKK7 activity is increased following phosphorylation at Ser and Thr residues within a Ser–Xaa–Ala–Lys–Thr (S–X–A–K–T) motif in the T-loop by multiple MAP3Ks. Similarly to MKK3 and MKK6, the amino acid following the Ser residue in MKK4 is a hydrophobic residue (Ile), whereas in MKK7, it is a basic residue (Lys). This distinction may, at least in part, account for the differential substrate specificities exhibited by certain MAP3Ks toward the MKK4 and MKK7 [26]. For instance, MEKK1 phosphorylates both MKK4 and MKK7, whereas MEKK4 selectively activates MKK4. This specificity is primarily mediated by a C-terminal DVD domain of approximately 24 amino acids in MKK4 and MKK7. Association with upstream kinases via DVD contributes to the conformational changes in the activation loop of MKK proteins, so that the Ser and Thr residues within the activation motif become accessible for efficient phosphorylation [20]. Beyond kinase interactions, the MKK4 DVD also binds scaffold proteins such as JIP-3 and POSH, forming stress-responsive signaling complexes [65,66].

Structurally, MKK4 adopts an inactive, autoinhibited conformation and may exist as a dimer. This conformation restricts access to the ATP-binding pocket and is destabilized upon phosphorylation [67]. In contrast, MKK7 exhibits significant plasticity in its catalytic domain. Crystal structures have captured MKK7 in multiple inactive states, including one where the hinge region Leu214 is in an unprecedented DFG-in/Leu-in conformation, and one where it assumes an inactive “out” DFG conformation [68,69]. Furthermore, the transition to the active state in MKK7 involves an N-terminal regulatory helix that acts allosterically to stabilize the active conformation, a mechanism likely shared across the MAP2K subfamily [70].

## 4. MKK4 and MKK7 Inhibitors

### 4.1. MKK4 Inhibitors

**Figure 5:**
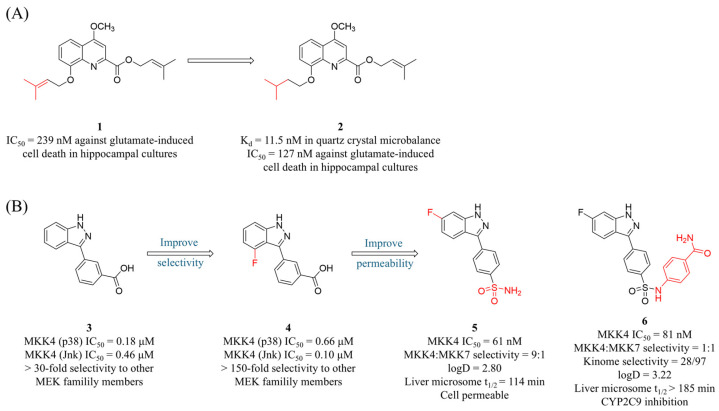
(**A**) Structures, affinities, and cellular activities of prenylated quinolinecarboxylic acid-derived MKK4 Inhibitors **1** and **2**. (**B**) Structures, inhibitory activities, and selectivity of 3-arylindole-derived MKK4 inhibitors **3**–**6**.

Given the structural features discussed above, particularly the conservation of the ATP pocket, achieving selectivity has been a primary challenge in MKK4 inhibitor design. Furthermore, structure-based design is challenging because the available MKK4 crystal structures (PDB: 3ALN, 3ALO, 3OUT) do not allow for the establishment of a reliable docking method. This may be due to inconsistencies in the ATP-binding pocket or the absence of co-crystallized ligands. Therefore, the development of selective MKK4 inhibitors often requires detailed SAR research. To develop MKK4 inhibitors with BBB penetration capability, Ogura et al. designed and synthesized a series of prenylated quinolinecarboxylic acid (PQA) derivatives based on **1** (Figure 5A), a secondary metabolite derived from *Polysphondylium pseudocandidum* [72]. Screening using a hippocampal neuronal glutamate toxicity model revealed that **2** (Figure 5A) exhibited the strongest neuroprotective activity (IC_50_ = 127 nM). In cellular models of Parkinson’s diseases (PD), Alzheimer’s disease (AD), and glutamate-induced cell death, **2** significantly inhibited caspase-3 activation induced by glutamate, MPP^+^, and amyloid-beta (Aβ), while also reducing the expression levels of p-JNK and p-MKK4. Furthermore, **2** selectively bound to the resting MKK4 (K_d_ = 11.5 nM) rather than activated MKK4. Further in vitro kinase assays confirmed that it inhibited the autophosphorylation of MKK4 at Thr2661 by competitively blocking the binding of sphingosine to MKK4. In a MPTP-induced neurodegenerative mouse model, treatment with **2** restored the number of tyrosine hydroxylase (TH)-positive neurons in the substantia nigra. Notably, compound **2** was rapidly detected in brain tissue within four hours of intraperitoneal administration, indicating its effective blood–brain barrier (BBB) penetration.

Leveraging the previously established MEK kinase screening platform, Scheidt’s group identified lead compound **3** (Figure 5B) featuring a 3-arylindazole scaffold [73]. Compound **3** competitively bound to the ATP-binding site of MKK4 and, notably, stabilized both its active and inactive forms to a similar extent. Subsequent structure–activity relationship (SAR) studies showed that modifications at the 5- and 6-positions of the indazole ring significantly enhanced inhibitory activity, with the 6-fluoro-substituted derivative **4** (Figure 5B) displaying the optimal activity. Notably, the N–H group in the indazole ring and carboxylic acid moiety were crucial for maintaining activity. Selectivity evaluation indicated that **4** exhibited more than 150-fold selectivity over other MEK family members but displayed a broad inhibition profile in kinome screening (S(35) = 0.21). Cellular studies revealed compound **4** failed to suppress cancer cell migration in a wound healing assay, a phenotype likely attributable to reduced membrane permeability due to the highly polar carboxyl group. To resolve this, Scheidt’s group employed a bioisostere strategy to structurally modify the carboxylic acid group and successfully obtained the para-substituted sulfonamide derivative **5** and **6** (Figure 5B) [74]. In CD18 and MiaPaCa2 pancreatic cancer cell lines, both **5** and **6** demonstrated significant antiproliferative activity and upregulated phosphorylation level of ERK1/2. In addition, co-administration of **5** with the MEK1/2 inhibitor significantly enhanced antiproliferative effects, achieving a coefficient of drug interaction (CDI) of 0.6. However, compound **6** exhibited significantly broader kinase inhibition (28/97 kinases at 10 μM) and reduced selectivity within the MEK family.

**Figure 6:**
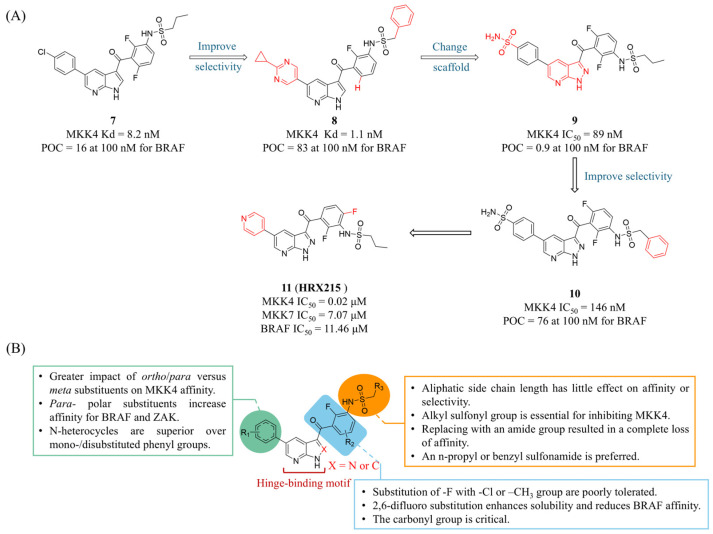
(**A**) The structures and inhibitory activities of MKK4 inhibitors **7**–**11**. (**B**) The SAR diagram of MKK4 inhibitors featuring a 7-azaindole or 1H-pyrazolo[3,4-*b*]pyridine scaffold.

Considering the off-target activity of Vemurafenib (**7**, the FDA-approved BRAF^V600E^ inhibitor, Figure 6A) towards MKK4, Laufer’s group employed an iterative multi-parameter optimization approach to systematically investigate the impact of key structural domains within **7** on the affinity and selectivity toward MKK4 [75]. The optimization process was based on binding affinity assessments using KINOMEscan technology, with data expressed as percentage of control (POC) values (where POC = 0 indicates complete binding, and POC = 100 indicates no binding). The final compound **8** (Figure 6A) exhibited improved MKK4 affinity and kinase selectivity, inhibiting only 5 out of 97 kinases with ≥65% inhibition at 1 μM, and none with ≥90% inhibition. In 2021, Laufer’s group replaced the 7-azaindole moiety in **7** with 1H-pyrazolo[3,4-*b*]pyridine and conducted a systematic SAR analysis (Figure 6B) [76]. Compound **9** (Figure 6A), featuring a para-sulfonamide group, was selected as a new lead compound due to its high affinity for MKK4. Attempts to further enhance selectivity for BRAF by introducing nonpolar substituents at the ortho position were unsuccessful. The authors then shifted their focus to modifying the sulfonamide side chain on the opposite side. The introduction of a benzyl group resulted in compound **10** with preserved activity while only inhibiting two kinases (AURKB and SNARK, POC < 35%) in a 97-kinase panel under 1 μM concentration.

In 2024, Laufer, Nyberg and Zender’s group reported the preclinical phase I clinical studies of first-in-class MKK4 inhibitor HRX215 (**11**, Figure 6A), which incorporates 2,6-difluorophenyl and pyridine moieties and exhibits improved water solubility and >100 selectivity against JNK1, BRAF, and MKK7 [21]. In preclinical studies, **11** demonstrated a significant pro-regenerative effect on the liver. Administering **11** (10 mg) to mice 12 h before and immediately prior to partial hepatectomy increased hepatocyte proliferation 5.3-fold (26.3% vs. 4.93% in the control group), an effect that remained effective even in fibrotic livers. Notably, no induction of hepatocyte proliferation or increase in liver-to-body weight ratio was observed when **11** was administered to wild-type mice, indicating that activation of stress signaling pathways in hepatocytes is required to unlock regenerative potential. Similarly, in a porcine model subjected to 80% hepatectomy, 43 h post-surgery the regeneration index (RI) in the **11** pre-treatment group was 21% higher than in the control group (2.57 vs. 2.12), and the regenerated liver volume increased by 57% (251.8 mL vs. 160.0 mL). In cases of fatal 85% hepatectomy, the survival rates in the **11** pre-treatment and post-treatment groups (66.7% [4/6], 83% [5/6]) were significantly higher than in the control group (17% [1/6]). Surprisingly, **11**-treated pigs did not exhibit increased intracranial pressure post-surgery, and the treatment prevented elevations in plasma ammonium, bilirubin, and hepatic aspartate aminotransferase. Additionally, **11** exhibited notable hepatoprotective effects. In a CCl_4_-induced liver injury model, **11** (0.4 mg/kg) significantly reduced apoptotic cells. Notably, it also demonstrated anti-steatotic and anti-fibrotic efficacy in models of alcoholic steatohepatitis (ASH) and chronic CCl_4_-induced liver fibrosis, respectively. The authors also investigated its inhibitory effects on HCC. In a mouse NASH-HCC model, **11** neither promoted tumorigenesis nor accelerated the progression of established liver tumors compared to the control group. Furthermore, it showed a significant anti-steatotic effect and a trend toward anti-fibrotic activity. Unlike the pro-regenerative signaling induced by MKK4 inhibition in hepatocytes, MKK4 inhibition in HCC cells led to significant downregulation of MKK7 and JNK1 along with strong activation of p38, followed by downregulation of Cyclin B1/D1/A2 and subsequent cell cycle arrest. In a Phase I clinical trial (EudraCT 2021-000193-28), a single or multiple dose administration (5–500 mg) of **11** to 48 healthy male volunteers resulted in an overall adverse event rate comparable to that of the placebo group (36.1% vs. 41.7%), with no severe adverse reactions observed. Pharmacokinetic (PK) analysis revealed dose-dependent absorption of compound **11**, and bioavailability was increased 2.1-fold when administered after a meal.

**Figure 7:**
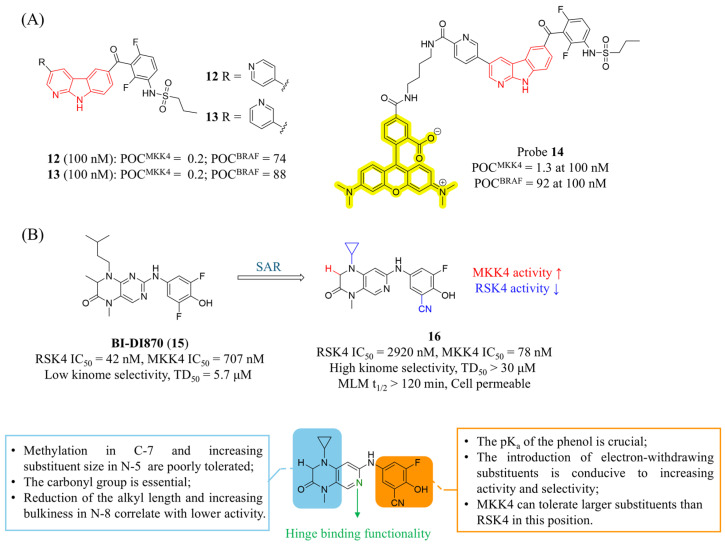
(**A**) The structures and inhibitory activities of MKK4 inhibitors **12**, **13** and **14**. (**B**) The structures, inhibitory activities, selectivity and SAR studies of compounds **15** and **16** featuring a 1,4-dihydropyrido[3,4-*b*]pyrazin-3(2H)-one scaffold.

In 2022, Laufer’s group also demonstrated that α-carboline can replace the 7-azaindole in **7** as a novel scaffold to drive selectivity [77]. The increased steric demand and its rigid structure proved to be a highly robust selectivity-inducing structural element without compromising potency. Representative compounds exhibited favorable profiles: compound **12** (Figure 7A) was non-cytotoxic in HepG2 cells (10 μM, 72 h), and compound **13** demonstrated high kinase selectivity across 320 kinases (S(20) = 0.103 at 100 nM). The authors propose that the rigid structure of α-carboline may occupy the hydrophobic pocket of MKK4 while creating steric hindrance with off-target kinases such as BRAF and ZAK. In summary, α-carboline supports the incorporation of various activity-enhancing groups without sacrificing selectivity, offering broad flexibility for PK optimization. Additionally, Laufer’s group designed novel fluorescent labeling compounds based on the α-carboline scaffold for high-throughput fluorescence polarization (FP) screening of MKK4 [78]. In the crystal structures of **7** binding to BRAF, the *para*-chlorophenyl group extends toward the solvent-accessible region, providing a proper site to link a fluorophore for avoiding steric clashes. Thus, a 5-TAMRA fluorophore was connected to the para-chlorophenyl group via carbon chains of varying lengths. The resulting probe **14** (Figure 7A) exhibited significantly enhanced binding to MKK4 and almost no binding to BRAF. In FP competition assays, probe **14** (20 nM) was able to distinguish MKK4 inhibitors with different affinities, suggesting its potential as a high-throughput screening (HTS) probe.

Recently, Laufer’s group reported the first highly selective 1,4-dihydropyrido[3,4-*b*]pyrazin-3(2H)-one MKK4 inhibitor [79]. This study was based on BI-D1870 (**15**, Figure 7B), a reversible ribosomal S6 kinase (RSK) inhibitor with broad off-target activity. After a systematic SAR exploration, the resulting compound **16** only inhibited three kinases at a concentration of 1 μM, significantly outperforming BI-D1870. It also showed excellent selectivity for RSK4 (MKK4 IC_50_ = 78 nM, RSK4 IC_50_ = 2920 nM, SI = 37.4). The thermal shift assay (TSA) and cellular thermal shift assay (CETSA) results confirmed the strong binding affinity of compound **16** to MKK4. In mouse HCC and human non-small cell lung cancer (NSCLC) cells, compound **16** displayed no significant cytotoxicity within the tested concentration range (IC_50_ > 30 μM), a feature attributable to its markedly reduced off-target activity. Furthermore, after incubation in mouse liver microsomes (MLMs) for 2 h, compound **16** did not undergo metabolic degradation, indicating favorable drug-like properties.

### 4.2. MKK7 Inhibitors

#### 4.2.1. Covalent Inhibitors

**Figure 8:**
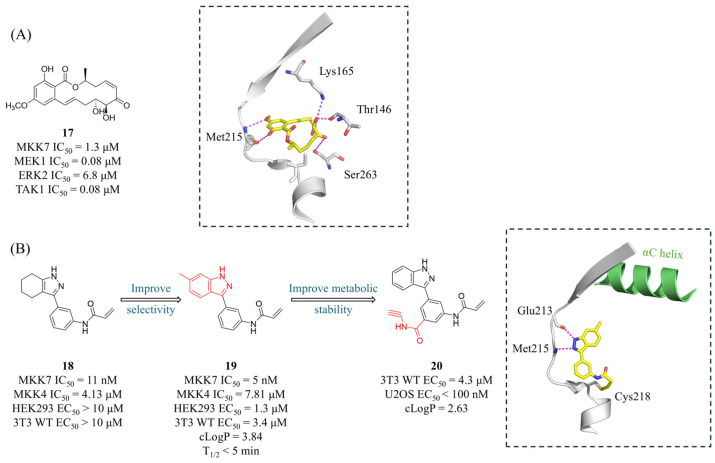
(**A**) The structure and inhibitory activities of MKK7 covalent inhibitor **17**. The dashed box indicates its co-crystal structure with MKK7 (PDB: 3wzu). (**B**) The structures and inhibitory activities of covalent inhibitors **18**, **19** and **20**. The dashed box indicates the co-crystal structure of **19** and MKK7 (PDB: 5z1d). Key amino acids are shown as sticks, with hydrogen bonds depicted as magenta dashed lines.

Unlike MKK4, the specific ligands for MKK7 are predominantly targeted covalent kinase inhibitors (TCKIs) since it is the only MEK isoform that possesses a cysteine residue in the hinge region (Cys218) [80]. The fungal natural product 5(Z)-7-oxozeaenol (**17**, Figure 8A) is the first reported covalent inhibitor of MKK7 [64,81,82]. Compound **17** moderately inhibits MKK7 (IC_50_ = 1.3 μM) and its α,β-unsaturated ketone moiety covalently binds to the Sγ of Cys218, which is located at the end of the hinge region. Even in the MKK7–C218S mutant, **17** does not form a covalent bond with Cys276, the gatekeeper cysteine adjacent to the DFG motif, likely due to steric hindrance by Met212. Additionally, the resorcinol moiety forms two hydrogen bonds with the backbone of hinge residue Met215. The two hydroxyl groups on the 14-membered ring also form hydrogen bonds with the backbone carbonyl groups of Thr146 and Ser263, as well as with the nitrogen atom of Lys165. Notably, there is a unique large hydrophobic pocket located behind the allyl group attached to the phenyl moiety.

London’s group conducted a virtual screening (VS) of a 117,667-compound acrylamide library with the aim of targeting the non-conserved Cys218 in MKK7 [83]. Candidates were required to form at least two hydrogen bonds with the kinase hinge region. The best hit, **18** (Figure 8B), exhibited potent inhibitory activity against MKK7 (IC_50_ = 11 nM), albeit with a micromolar range cellular activity in HEK293 and 3T3 cells. Based on the docking model, the authors optimized the lead compound using an indazole scaffold and found that substitutions were well tolerated at the 6-position but not at the 7-position. Although the resulting inhibitor **19** (Figure 8B) showed enhanced selectivity over MKK4, it was found to inhibit 4 out of 76 kinases in the panel (Aurora B, LRRK2, MKK4, and FLT3) by more than 75% at 1 μM, and it exhibited poor liver microsomal stability (t_1/2_ < 5 min). In addition to the covalent binding to Cys218, the indazole nitrogen mediated two hydrogen bonds in the hinge region in the crystal structure. Efforts to reduce lipophilicity and improve metabolic stability led to the discovery of compound **20** (Figure 8B), which showed good selectivity in proteomic profiling of bacterial and human cell lysates (MDA-MB-231 cells) but also displayed some cytotoxicity. In an assay blocking activation of primary mouse B Cells, **19** (10 μM) suppressed LPS-induced CD86 expression (a marker of B-cell activation) by 60%, whereas **20** achieved 90% inhibition.

**Figure 9:**
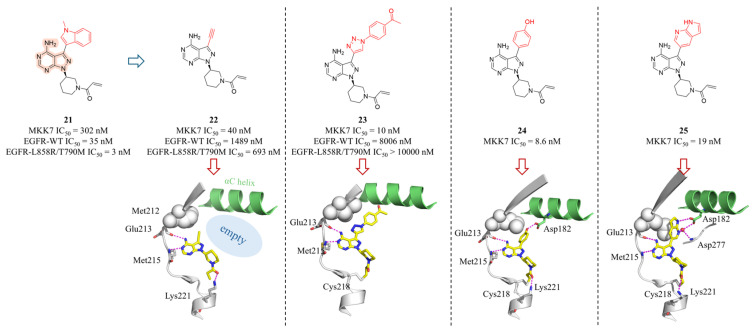
The structures of compounds **21–25**, along with their inhibitory activities against targets and cells. The co-crystal structures are located below each compound: **22** (PDB: 6ib0), **23** (PDB: 6ib2), **24** (PDB: 6qg4), and **25** (PDB: 6qhr). The α-C helix of MKK7 is shown in green, with interacting residues represented as sticks and hydrogen bonds depicted as magenta dashed lines.

Due to the structural similarity between EGFR and MKK7, Rauh’s group designed a series of MKK7 covalent inhibitors based on the pyrazolopyrimidine scaffold of EGFR inhibitor **21** (MKK7 IC_50_ = 302 nM) [68]. Replacing the indole group in **21** with an alkyne significantly enhanced both activity and selectivity (**22**, IC50 = 40 nM). The co-crystal structure of **22** with MKK7 revealed additional space between the Met212 gatekeeper in the MKK7 back pocket and the catalytic Lys165 (Figure 9). Utilizing the alkyne for copper-catalyzed click chemistry to introduce a *para*-ketophenyl derivative (**23**) markedly improved activity and selectivity against EGFR. Additionally, the authors employed Robotics-Assisted Screening Platform for Efficient Ligand Discovery (RASPELD) to conduct a focused screening of 101 EGFR-targeted compounds [84]. Hit **24** (with a para-phenolic group) and **25** (with a pyrrolo[2,3-*b*]pyridine substitution) exhibited excellent MKK7 inhibitory activity (Figure 9). SAR studies highlighted the importance of covalent inhibition, as non-covalent or reversibly covalent inhibitors lacking the acrylamide warhead showed no activity. The covalent inhibitors **23**, **24**, and **25** exhibited time-dependent engagement with MKK7, a process significantly delayed by ATP competition. Their crystal structures elucidated the binding mode, revealing two key interactions: the pyrazolopyrimidine core forms conserved hydrogen bonds with Glu213 and Met215, while the acrylamide warhead covalently binds Cys218 and its carbonyl group hydrogen-bonds with Lys221. Differently, the phenolic hydroxyl group of **24** forms an additional hydrogen bond with Asp182, and the nitrogen atoms of the pyrrolopyridine in **25** interacts with the hinge region, explaining their enhanced activity. Notably, although the binding modes of these compounds were similar to those observed for EGFR inhibitors, the orientation of the flexible methionine gatekeeper side chain (Met212 in MKK7) was slightly different. A kinase selectivity profile across 320 kinases showed that **23** at 1 μM inhibited only seven off-target kinases (BLK, BMX, BTK, ITK, JAK3, mTOR, S6K), all of which contain a conserved Cys residue. In cellular assays, **23** completely suppressed the phosphorylation of JNK and c-Jun. Compound **23** exhibited low lipophilicity (cLogP = 1.78), poor aqueous solubility (8 μM), but favorable ligand lipophilic efficiency (LLE = 6.22). It demonstrated good drug absorption in Caco-2 permeability assays and exhibited stability in human and mouse plasma, albeit with moderate microsomal stability.

**Figure 10:**
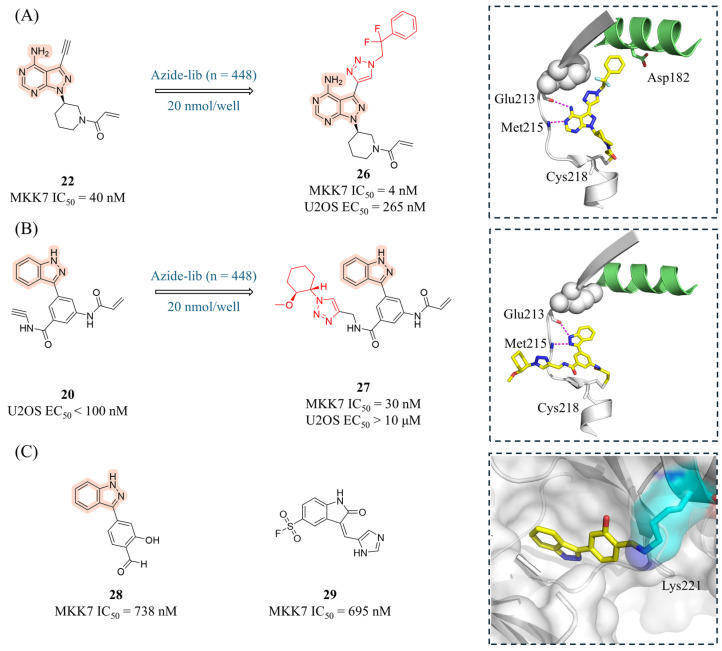
(**A**) The CuAAC reaction for LSF of compound **22**. The dashed box shows the co-crystal structure of **26** with MKK7 (PDB: 7ovj), where Asp182 is displaced due to steric hindrance. (**B**) The CuAAC reaction for LSF of compound **20**. The dashed box shows the co-crystal structure of **27** with MKK7 (PDB: 7ovl) with interacting residues represented as sticks and hydrogen bonds depicted as magenta dashed lines. (**C**) The structures and inhibitory activities of Lys221-targeting covalent compounds **28** and **29**. The dashed box shows the co-crystal structure of **28** with MKK7 (PDB: 9hz0).

In 2022, Rauh and London’s group employed the robust copper(I)-catalyzed alkyne–azide cycloaddition (CuAAC) reaction for late-stage functionalization (LSF) at the nanomolar scale in a plate-based format [85]. This workflow is compatible with efficient large-scale cellular and biochemical assays for high-throughput testing of covalent inhibitors. The authors selected compound **20** and **22** as starting points for the workflow, reacting them with a library of 448 commercially available azides via CuAAC. The In-Cell Western assay (ICW) was subsequently used to perform high-throughput detection of the phosphorylation of JNK by the products. The most potent derivative **26** (Figure 10A) demonstrated superior target inhibition and intracellular activity (IC_50_ = 4 nM, EC_50_ = 265 nM) compared to **22**, likely driven by additional lipophilic interactions within the MKK7 back pocket. In contrast, compound **27** (derivative of **20**, Figure 10B) showed significantly reduced activity at both the protein and cellular levels. However, targeting the solvent-exposed region also offers hope for the future design of potential exit vectors, serving as a blueprint for the design of PROTACs or other bivalent compounds. Co-crystal structures revealed that the triazole moiety did not form additional interactions either within the back pocket (for **22** derivatives) or in the solvent-exposed periphery of the binding site (for **20** derivatives). Interestingly, Asp182 is pushed away in the **26**/MKK7 complex to accommodate the extended compounds. Compared to **22**, **26** exhibited better kinetic solubility (174.1 μM), reduced efflux, similar PAMPA and plasma stability values (Plasma stability human/mouse = 116/94% remaining) but poorer intestinal permeability (Caco-2 *P*_app_(A − B) = 11.81 × 10^−6^ cm/s), and HLM stability (CL_int_ = 308.1 nL/min/mg).

While covalent drugs offer advantages like high potency, most have historically targeted the cysteine residue. Lysine is an attractive alternative target due to its abundance, but its high flexibility and different chemical properties (like pK_a_) make it difficult to design binders. Recently, Rauh and London’s group presented two new computational methods, developed within the Rosetta software framework, for designing lysine-targeting covalent inhibitors [86]. In a “ligand-side” approach, a virtual library of “electrophilic analogs” is created by adding different warheads to different positions on the ligand and is subsequently docked into the target protein. In a “protein-side” approach, an electrophile is installed on the target lysine and model its conformational space to find suitable installation vectors on the ligand. Both of these protocols were applied retrospectively to a data set of electrophilic ligands and to a data set of vitamin B6 covalently bound to a receptor lysine residue. The ligand-side protocol successfully identified the known covalent binder in 80% and 86% of cases, while the protein-side protocol achieved identification rates of 56% and 82%, respectively. In the context of the MKK7 kinase, both protocols predicted that compound **28** (a derivative of **18**) and **29** would be capable of targeting Lys221 (Figure 10C). Compounds **28** and **29** were synthesized and successfully inhibited MKK7, with IC_50_ values of 738 nM and 695 nM, respectively. Mass spectrometry confirmed that compound **28** formed a reversible covalent bond with MKK7. The co-crystal structure of compound **28** with MKK7 (PDB: 9hz0) confirmed Lys221 as the binding site. Although the electron density for the covalent linkage was poor, the structure revealed a large movement of the lysine side chain.

**Figure 11:**
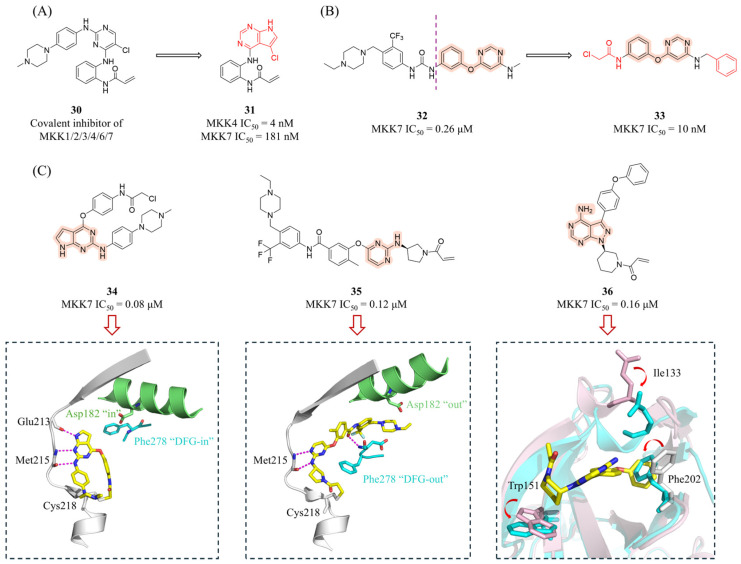
(**A**,**B**) The structures and inhibitory activities of covalent inhibitors **30**–**33**. (**C**) The structures of compounds **34**–**36**, along with their inhibitory activity against MKK7 and co-crystal structures (PDB IDs from left to right: 6yg3, 6yg6, and 6yz4). Key amino acid residues are represented as sticks, and hydrogen bonds are depicted as magenta dashed lines. The inset of ibrutinib shows structural superposition with the apo structure (pink), revealing the conformational rearrangement required for pocket formation (indicated by arrows).

Jiang et al. employed a structural modification strategy to optimize the pan-kinase inhibitor **30** [87] into a covalent inhibitor targeting the conserved cysteine residues (Cys247 in MKK4 and Cys261 in MKK7) [62]. This design retained the acrylamide warhead while replacing the pyrimidine core with a pyrrolo[2,3-*d*]pyrimidine scaffold. This modification maintained dual hydrogen-bonding interactions with the hinge region (Met181/Glu179) in MKK4 and simultaneously optimized the spatial proximity between the acrylamide and the target cysteine. The reversible binding affinity in **30** was eliminated by removing the phenylpiperazine moiety. Further introduction of a chlorine atom at the 5-position of the pyrrolopyrimidine core ultimately yielded the potent covalent inhibitor **31** (Figure 11A, MKK4 IC_50_ = 4 nM; MKK7 IC_50_ = 181 nM). Competitive pull-down assays demonstrated that 5 μM **31** effectively blocked the binding of the biotinylated probe to MKK4. The pan-kinome profiling revealed that in MDA-MB-231 cells, this compound selectively occupied MKK4 (90.2%) and MKK7 (87.0%) with no impact on other kinases. Treatment with 10 μM **31** completely inhibited anisomycin-induced JNK phosphorylation without affecting the phosphorylation levels of p38, ERK1/2, or ERK5, confirming its favorable pathway selectivity. Notably, even in MKK4- or MKK7-knockdown cells, **31** still fully blocked JNK phosphorylation, an effect dependent on its covalent binding properties. Although the compound alone did not significantly inhibit the proliferation of MDA-MB-231 cells, it exhibited strong synergistic effects when combined with a JNK inhibitor (CI = 0.7–0.9). Recently, Okada’s group reported that at concentrations that did not affect the viability of normal fibroblasts, **31** effectively inhibited JNK activity and reduced the expression of stem cell markers in glioma stem cells (GSCs) [88]. Furthermore, the capacity of GSCs that survived the **31** treatment to form spheres and initiate tumors was impaired.

Scheidt’s group employed sub-micromolar off-target inhibition of MKK7 by the FMS-like tyrosine kinase 3 (FLT3) inhibitor AST-487 (**32**, Figure 11B) and designed novel covalent MKK7 inhibitors through covalent modifications [89]. After truncating the structure of **32** to retain the phenoxypyrimidine core and introducing an α-chloroacetamide as a covalent warhead targeting Cys218, they explored the chemical space around the aminopyrimidine moiety. Among the derivatives, benzylamine derivative **33** (Figure 11B) exhibited the highest potency (IC_50_ = 10 nM), while N-methylation or ortho-substitution significantly reduced activity. Although compound **33** showed no activity against MKK3/4/5/6, it displayed notable binding to EGFR and EGFR-L858R in a 97-kinase screening panel. In T-cell acute lymphoblastic leukemia (T-ALL) cell lines, compound **33** demonstrated potent cytotoxicity within 48 h (EC_50_ = 1.1–2.9 μM), and the cytotoxic effect persisted after compound removal, supporting an irreversible inhibition mechanism.

Schröder et al. used melting thermal shift (ΔTm) assay to screen an in-house library of 360 compounds against MKK7 [70]. These efforts identified nine Type I and Type II covalent inhibitors, among which the Type I inhibitor **34** (Figure 11C) exhibited an IC_50_ value of 0.08 μM against MKK7. The Type II compound **35** (Figure 11C) showed the highest thermal shift of 24.7 °C and displayed similar activity (IC_50_ = 0.12 μM). Compounds **34** and **35** significantly suppressed the levels of phosphorylated JNK and c-Jun in sorbitol-treated THP-1 cells at nanomolar concentrations. Co-crystal structures revealed that **34** forms a covalent bond with Cys218 and establishes three hydrogen bonds through its 2-aminopyrolopyrimidine and aminopyrazole scaffolds, thereby stabilizing the DFG-in conformation. In contrast, **35** induces an outward movement (~23°) of the αC-helix and promotes a DFG-out conformation by covalently binding to Cys218, creating a hydrophobic pocket required for its trifluoromethylphenyl moiety. Unexpectedly, ibrutinib (**36**, Figure 11C) was found to bind not only in the ATP pocket but also at an allosteric site located at the top of the kinase N-lobe. Significant structural alterations for the pocket formation included an “out” swing of Phe202, an outward movement of the helical turn preceding the β1 that harbors Ile133 as well as a small alteration of the Trp151 side chain. Isothermal titration calorimetry (ITC) confirmed that this binding is independent of the ATP site, providing a new strategy for the development of allosteric Type III inhibitors.

#### 4.2.2. Other Inhibitors

**Figure 12:**
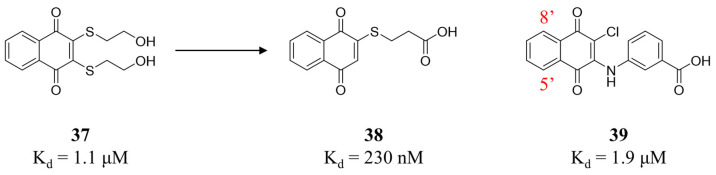
Structures and target affinity of the reversible MKK7 inhibitors **37–39**.

Given that naphthoquinone **37** is a reversible weak inhibitor of MKK7 (K_d_ = 1.1 μM) [90], Quinn’s group conducted a systematic SAR study of natural and synthetic naphthoquinone derivatives (Figure 12) [91]. Among the 33 naphthoquinone compounds evaluated, compounds **38** and **39** exhibited the highest affinities (**38**: K_d_ = 230 nM; **39**: K_d_ = 1.9 μM) and showed no activity against MKK4. The SAR analysis revealed that the length of the sulfur-containing side chain is critically important for activity—compound **38** (with a propionic acid chain) displayed greater potency than those with an acetic acid or isobutyric acid chain. Additionally, methoxy substitution or methoxylation at the 5,8-positions of the naphthoquinone ring significantly reduced activity. Compounds **38** and **39** demonstrated antiproliferative activity against various tumor cell lines. Docking studies showed that the thio-propionic acid chain of **38** formed hydrogen bonds with Lys165 and Asp277, while the quinone carbonyl group interacts with Cys276 and Met215.

DTP3 (Ac-D-Tyr-D-Arg-D-PheNH_2_, **40**) is a D-configured tripeptide identified through the screening of a combinatorial library of synthetic peptides targeting the GADD45β/MKK7 complex [49]. In multiple myeloma (MM), NF-κB upregulates GADD45β, which binds to MKK7 and blocks its enzymatic activity, thereby suppressing JNK-mediated apoptosis. DTP3 specifically binds to MKK7 with high affinity, inducing a conformational rearrangement that effectively displaces GADD45β. This “relieves” the inhibition of MKK7, allowing it to activate the JNK signaling cascade and trigger cancer-cell-selective apoptosis. Computational and NMR studies indicate that DTP3 interacts with a shallow pocket on the N-terminal region of MKK7, located proximally to the ATP pocket. The aromatic side chains (D-Tyr and D-Phe) are the primary drivers of this interaction, while the central D-Arg residue contributes minimally [50,92]. DTP3 is highly selective for cancer cells because the GADD45β/MKK7 survival module is non-redundant in these cells, whereas most normal cells do not require GADD45β for survival. In preclinical mouse models, systemic administration of DTP3 induced complete regression of established MM xenografts. In addition, DTP3 has shown the capacity to synergize with bortezomib and can bypass drug resistance to conventional treatments like steroids and immunomodulatory agents [93]. DTP3 exhibits linear systemic exposure, a long terminal plasma half-life (approximately 11–19 h in preclinical species), and good bioavailability. It distributes extensively to most tissues but notably does not cross the blood–brain barrier. Currently, DTP3 has advanced into Phase I/IIa clinical trials for patients with relapsed or refractory MM, where initial results confirmed its ability to induce a cancer-selective pharmacodynamic response without adverse side effects.

A comprehensive summary of the representative MKK4 and MKK7 inhibitors discussed in this review, including their chemical scaffolds, mechanisms of action, and therapeutic potential, is provided in Table 2.

## 5. Conclusions and Future Perspectives

As elucidated in this review, the strategic positioning of MKK4 and MKK7 as the “gatekeepers” of the JNK signaling architecture offers a unique therapeutic opportunity to modulate maladaptive stress responses with greater precision than direct JNK inhibition. While both kinases share high sequence identity and a canonical bilobal fold, their distinct physiological roles—MKK4 as a regenerative brake and MKK7 as an inflammatory driver—have necessitated divergent medicinal chemistry strategies.

For MKK4, the field has witnessed a paradigm shift from oncology-focused exploration to regenerative medicine. The successful advancement of HRX215 into clinical trials marks a pivotal milestone, providing the first human proof-of-concept that pharmacological inhibition of MKK4 is safe and effective for unlocking liver regeneration. From a structural perspective, the evolution of scaffolds highlights the triumph of structure-based drug design in overcoming the “selectivity challenge” against p38 MAPK and BRAF. Future efforts in this domain are required to refine physicochemical properties to balance liver accumulation with systemic safety.

In contrast, the development of MKK7 inhibitors has been defined by the exploitation of the rare Cys218 residue within the hinge region. This unique structural feature has enabled the design of highly potent covalent inhibitors that offer prolonged target residence time, a property particularly advantageous for treating chronic fibrotic and inflammatory conditions. Moreover, the application of advanced techniques like LSF via click chemistry showcases the power of modern chemical biology in accelerating inhibitor development.

Looking forward, several frontiers remain to be explored. First, while ATP-competitive inhibitors dominate the current landscape, the development of allosteric inhibitors—as hinted by the binding mode of ibrutinib—could offer superior specificity by targeting non-conserved pockets. Second, the application of proteolysis-targeting chimeras (PROTACs) remains an untapped potential for MKK4/7. Given the solvent-exposed exit vectors identified in recent SAR studies (e.g., the para-position of phenyl rings in MKK4 inhibitors), designing bifunctional degraders could eliminate the scaffolding functions of these kinases, potentially yielding distinct biological outcomes compared to kinase inhibition alone. Furthermore, the emerging class of dual MKK4/7 covalent inhibitors offers a rational combination strategy for aggressive cancers where pathway redundancy limits the efficacy of single-target agents.

In terms of clinical application, while first-generation MKK4/7 inhibitors like HRX215 are primarily intended for acute indications (e.g., liver regeneration), the development of inhibitors for chronic diseases such as fibrosis and cancer necessitates the anticipation of acquired resistance. Drawing from the history of kinase inhibitors, two primary mechanisms are theoretically predictable. First, for covalent MKK7 inhibitors, the reliance on the non-catalytic Cys218 residue presents a specific vulnerability. Analogous to the C481S mutation in BTK inhibitors (e.g., ibrutinib) [94] or the C797S mutation in EGFR inhibitors (e.g., osimertinib) [95], chronic exposure may select for Cys218Ser or Cys218Ala mutations. Such mutations would abolish the covalent anchorage, significantly reducing the potency of irreversible inhibitors, thereby necessitating the development of next-generation reversible or allosteric binders. Second, adaptive pathway rewiring represents a broad challenge. The MAPK signaling network is characterized by extensive feedback loops and crosstalk. As discussed in Section 2.2, MKK4 inhibition can unleash the “brake” on the p38 MAPK pathway due to the loss of negative feedback or competition for upstream MAP3Ks. In a tumor context, this compensatory activation of p38 or the upregulation of parallel survival pathways (e.g., PI3K/Akt) could serve as a bypass mechanism, limiting the long-term efficacy of monotherapy.

To counteract these adaptive resistance mechanisms and enhance therapeutic outcomes, the field is shifting towards rational combination strategies. Current preclinical data support three synergistic approaches: (1) Horizontal Blockade: Co-targeting parallel MAPK pathways can prevent compensatory signaling. For example, combining the MKK4 inhibitor Compound **5** with MEK1/2 inhibitors has demonstrated significant synergy in *KRAS*-mutant pancreatic cancer models. (2) Vertical Inhibition: Simultaneous targeting of multiple nodes within the same cascade (e.g., using the dual MKK4/7 inhibitor Compound **31** with downstream JNK inhibitors) ensures complete pathway abrogation in JNK-driven tumors. (3) Chemosensitization: MKK7 inhibition can lower the apoptotic threshold; notably, the PPI inhibitor DTP3 sensitizes multiple myeloma cells to bortezomib, overcoming proteasome-inhibitor resistance. These strategies suggest that future clinical regimens will likely position MKK4/7 inhibitors as potent adjuvants rather than standalone agents.

In summary, the medicinal chemistry of MKK4 and MKK7 has matured significantly over the past decade. As research transitions from the discovery of chemical probes to the validation of clinical candidates, these upstream kinases stand poised to transform the treatment landscape for acute liver failure, tissue fibrosis, and specific malignancies.

## Figures and Tables

**Table 1:** Summary of activation stimuli and mechanisms for major MAP3Ks.

MAP3Ks	External/Internal Stimuli	Mechanism
TAK1	TNF-α, IL-1, LPS, TGF-β, ROS, osmotic pressure	Sensing Lys63-linked polyubiquitinylation events at the TNF receptor. Activated through ubiquitination modifications by TRAF proteins.
ASK1–3	H_2_O_2_, ROS, ER stress, TNF-α, Fasl, calcium ion	Inhibited by binding to antioxidant proteins (such as Thioredoxin) in the resting state; oxidative stress causes its dissociation and activation.
MLKs	TNF-α, IL-1, UV, heat shock, osmotic pressure, GPCR signals	Involved in cytoskeletal rearrangement, activated by small GTPases (such as Rac1, Cdc42).
MEKK1–4	MEKK1: UV, serum deprivation, cytoskeletal perturbation MEKK2/3: TNF-α, IL-1, LPS MEKK4: osmotic pressure, cytotoxic agent	Partially activated through recruitment by small GTPases (Rho, Rac) or receptor complexes.
DLK	neuronal damage (axotomy, ischemia), oxidative stress	Primarily expressed in the nervous system and involved in neurodegeneration and regeneration.
LZK	neuronal damage, oxidative stress	Functionally redundant with DLK, acting as a co-regulator of the JNK pathway in neurons.
HPK1	lymphocyte antigen receptors, TNF-α, IL-1, LPS	Requiring own phosphorylation on tyrosine and subsequent interaction with adaptors of the SLP family. Ubiquitinated by E3 ubiquitin ligases, leading to its proteasomal degradation and limited signal duration.

**Table 2:** Summary of key small-molecule inhibitors of MKK4 and MKK7.

Target	Representative Compounds	Scaffold/Chemical Class	Mechanism of Action	Key Features & Therapeutic Potential	Ref.
MKK4	Compound **2**	Prenylated quinolinecarboxylic acid (PQA)	ATP-competitive (Reversible)	BBB penetrant; Neuroprotection in AD/PD models.	[71]
	Compound **5**	3-Arylindazole	ATP-competitive (Reversible)	Improved cell permeability; Synergistic with MEK inhibitors in pancreatic cancer.	[73]
	HRX215 (**11**)	1H-pyrazolo[3,4-*b*]pyridine	ATP-competitive (Reversible)	First-in-class clinical candidate; High selectivity against BRAF; Promotes liver regeneration.	[21]
	Compound **13**	α-Carboline	ATP-competitive (Reversible)	Rigid scaffold induces high kinome selectivity; Suitable for fluorescent probe design.	[76]
	Compound **16**	1,4-dihydropyrido[3,4-b]pyrazin-3(2H)-one	ATP-competitive (Reversible)	Derived from RSK inhibitors; Excellent selectivity and metabolic stability.	[78]
MKK7	5Z-7-Oxozeaenol (**17**)	Resorcinol lactone (Natural Product)	Covalent (Targeting Cys218)	First covalent MKK7 inhibitor; Moderate selectivity.	[63]
	Compound **20**	3-phenyl-1*H*-indazole	Covalent (Targeting Cys218)	Good selectivity but also displayed some cytotoxicity	[82]
	Compound **23**	Pyrazolopyrimidine	Covalent (Targeting Cys218)	Derived from EGFR inhibitors; Optimized via click chemistry; High LLE.	[67]
	Compound **28**	3-phenyl-1*H*-indazole	Covalent (Targeting Lys221)	Novel binding mode targeting the catalytic lysine; Designed by computational methods.	[86]
	Ibrutinib (**36**)	Pyrazolo[3,4-*d*]pyrimidine	Type III (Allosteric)/ATP-competitive	FDA-approved drug; Binds to a novel N-lobe allosteric site; Template for Type III inhibitor design.	[69]
	DTP3	D-tripeptide	PPI Inhibitor (Allosteric)	Disrupts GADD45β-MKK7 interaction; Clinical candidate for Multiple Myeloma.	[47]
Dual	Compound **31**	Pyrrolo[2,3-d]pyrimidine	Covalent (Targeting Cys247/Cys261)	Dual covalent inhibition; Synergistic effects with JNK inhibitors in cancer cells.	[61]

## Data Availability

Data sharing is not applicable to this article as no new data were created. The crystal structure data discussed in this review are available in the Protein Data Bank (PDB) under the accession codes cited in the text and figure legends.

## Abbreviations

The following abbreviations are used in this manuscript:

Aβ	amyloid-beta
AD	Alzheimer’s disease
ALF	severe acute liver failure
ATF2	activating transcription factor 2
ASH	alcoholic steatohepatitis
ASO	antisense oligonucleotides
BBB	blood–brain barrier
CETSA	cellular thermal shift assay
CLD	chronic liver diseases
CuAAC	copper(I)-catalyzed alkyne–azide cycloaddition
D domain	docking domain
DVD	domain for versatile docking
FLT3	FMS-like tyrosine kinase 3
FP	fluorescence polarization
GSC	glioma stem cell
HCC	hepatocellular carcinoma
HTS	high-throughput screening
ICW	In-Cell Western assay
ITC	Isothermal titration calorimetry
JIP1	JNK-interacting protein 1
JNK	c-Jun N-terminal kinase
KLF4	Kruppel-like factor 4
LLE	ligand lipophilic efficiency
LPS	lipopolysaccharides
LSF	late-stage functionalization
MAPK	mitogen-activated protein kinase
MAP2Ks	mitogen-activated protein kinase kinases
MLM	mouse liver microsomes
MMP	matrix metalloproteinase
NSCLC	non-small cell lung cancer
PD	Parkinson’s diseases
PK	pharmacokinetic
PLK4	Polo-like kinase 4
POC	percentage of control
POSH	plenty of SH3
PQA	prenylated quinolinecarboxylic acid
PROTACs	proteolysis-targeting chimeras
RA	rheumatoid arthritis
RASPELD	Robotics-Assisted Screening Platform for Efficient Ligand Discovery
RSK	ribosomal S6 kinase
RNAi	RNA interference
SAR	Structure–activity relationship
T-ALL	T-cell acute lymphoblastic leukemia
TCKIs	targeted covalent kinase inhibitors
TH	tyrosine hydroxylase
TSA	thermal shift assay
VS	virtual screening
